# Mitochondrial genomes of the bird genus *Piranga*: rates of sequence evolution, and discordance between mitochondrial and nuclear markers

**DOI:** 10.1080/23802359.2019.1637286

**Published:** 2019-07-16

**Authors:** Luke C. Campillo, Kevin J. Burns, Robert G. Moyle, Joseph D. Manthey

**Affiliations:** a Biodiversity Institute and Department of Ecology and Evolutionary Biology, University of Kansas, Lawrence, KS, USA;; b Biology Department, University of Hawai‘i – Mānoa, Honolulu, HI, USA;; c Department of Biology, San Diego State University, San Diego, CA, USA;; d Department of Biological Sciences, Texas Tech University, Lubbock, TX, USA

**Keywords:** Mito-nuclear discordance, mitogenome, tanager

## Abstract

We report the characteristics of the mitochondrial genomes of 22 individuals in the bird genus *Piranga*, including all currently recognized species in the genus (*n* = 11). Elements follow the standard avian mitogenome series, including two ribosomal RNA (rRNA) genes, 22 transfer RNA (tRNA) genes, 13 protein coding genes, and the mitochondrial control region. Excluding tRNA sequences, sequence divergence rate was lowest in rRNA genes and highest in genes encoding NADH (specifically ND1, ND2, ND3) and the control region. Gene trees assembled from 16 elements (non-tRNAs) varied greatly in topological concordance compared to the recognized species tree (based on thousands of nuclear loci), with no one gene tree precisely recovering all relationships in the genus. We also investigated patterns of concordance between the mitogenome tree and the nuclear species tree and found some discrepancies. Across non-tRNA gene trees (*n* = 16), the species tree topology was recovered by as few as three elements at a particular node and complete concordance (i.e. 16/16 gene trees matched the species tree topology) was recovered at only one node. We found mitochondrial gene regions that are often used in vertebrate systematics (e.g. CytB, ND2) recovered nearly the exact same topology as the nuclear species tree topology.

## Introduction

Mitochondrial DNA (mtDNA) has long been used to elucidate phylogenetic relationships across the tree of life. The ubiquity of mitochondrial sequence data in contemporary systematic research, ranging from single markers (Burbrink et al. 2016) to complete mitogenomes (Nguyen and Ho 2016), indicates the continued and pervasive utility of these markers. However, the distinctive transmission of mitochondrial genes (i.e. maternal inheritance and faster rates of fixation), the unique evolutionary history of nuclear genes and gene tree discordance (i.e. gene tree topologies different from one another or gene trees incongruous with the species phylogeny), due to incomplete lineage sorting (ILS) or deep coalescence (Maddison 1997), have proven to be particularly problematic for phylogenetic reconstruction (McGuire et al. 2007). Despite the recognized inherent problems that accompany phylogenetic reconstruction using mitochondrial and/or nuclear genes (reviewed by Toews and Brelsford 2012), mtDNA remains a valuable tool in any systematic toolkit.

In the bird genus *Piranga,* several studies have used mtDNA to investigate phylogenetic relationships (Burns 1998; Barker et al. 2015), but reconstructions varied in topology and nodal support depending on the mtDNA genes used (CytB or CytB + ND2, respectively). Recently, we used restriction-site associated DNA sequencing (RAD-seq; Miller et al. 2007) and target capture of ultraconserved elements (UCEs; Faircloth et al. 2012) to resolve the phylogeny of the genus, producing topologically identical species tree reconstructions with high support across nodes for both sequencing methods (Manthey et al. 2016), but different from previous, mtDNA-based phylogenies (i.e. Burns 1998; Barker et al. 2015).

Due to inherent features of target-capture sequencing methods, a by-product of sequencing UCEs is the potential to recover the full mitochondrial genome (mitogenome). Here, we utilize published target-capture sequence data to recover a set of complete mitogenomes for the genus *Piranga* to investigate patterns of concordance among individual mitochondrial gene trees, the mitogenome tree, and the nuclear species tree.

## Methods

We used published Illumina sequence data (Manthey et al. 2016; NCBI SRA BioProject PRJNA296706) to assemble mitochondrial genomes from 22 individuals in the genus *Piranga* and a single outgroup individual. Full details on the specimens used in this study, including collection locality and tissue accession numbers can be found in Manthey et al. (2016). Here, we list species names and their associated voucher specimen numbers: *P. bidentata* (MZFC 17737, MZFC 19257), *P. erythrocephala* (FMNH 343370, FMNH 343371), *P. flava* (KU 90809, LSU B15408), *P. hepatica* (KU 4970, KU 9084), *P. leucoptera* (FMNH 481795, LSU B7783), *P. ludoviciana* (SDSU 2385, SDSU 2650), *P. lutea* (KU 89864, LSU B5400), *P. olivacea* (KU 2699, KU 4672), *P. roseogularis* (KU 2049, KU 2141), *P. rubra* (KU 26572, KU 7046), *P. rubriceps* (LSU B265, LSU B34818), and *Cardinalis cardinalis* (KU 21828). All mitogenomes are accessioned in NCBI’s GenBank: MH700631-MH700653.

Bioinformatics were initially performed for the UCE project (Manthey et al. 2016), utilizing the PHYLUCE software package (Faircloth 2015) and Trinity (version: rnaseq_r2013_08_14; (Grabherr et al. 2011) to assemble contigs for each individual. Mitochondrial genomes, usually the longest one or two contigs of a UCE assembly, were extracted, aligned, and annotated with the guidance of the mitochondrial genome of one *Cardinalis cardinalis* individual (GenBank #NC025618) using Geneious v9.1.4 (https://www.geneious.com). For each of the elements (non-tRNA mitochondrial elements in Table 1), we estimated gene trees in RAxML with the GTR + Γ substitution model (Silvestro and Michalak 2011; Stamatakis 2014). Gene tree support was assessed with 200 rapid bootstrap replicates. All gene trees and phylip files have been uploaded to FigShare (10.6084/m9.figshare.6807386; 10.6084/m9.figshare.8141819, respectively).

We scored for topological concordance between individual gene trees and the concatenated mitogenome tree (also produced in RAxML, with 500 rapid bootstrap replicates) using the program PhyParts (Smith et al. 2015). We did this analysis twice, first with no limit on bipartition support (i.e. bootstrap value could be 0 on any given node in any individual gene tree) and then requiring at least 50% bootstrap support at a given node in a gene tree. Lastly, we calculated difference in likelihood scores (ΔML) between gene trees inferred with topology as a free parameter (i.e. standard ML gene tree inference) and those where topology was constrained to match the mitogenome tree. This allowed a formal assessment of changes in likelihood scores across the different partitions (i.e. genes; Walker et al. 2018).

## Results

The order of elements along each newly assembled mitochondrial genome was identical to the published *C. cardinalis* individual used as the reference genome. Mitochondrial genomes in *Piranga* ranged from 16,782 to 16,814 bp (mean = 16,804), with variation due to indels found in spacers or the mitochondrial control region. In protein-coding and rRNA genes, as well as the control region, nucleotide identity was conserved between 63 and 90% within *Piranga* (Table 1), and amino acid conservation in protein coding genes ranged between 79 and 98%. Nucleotide sequence and amino acid residue conservation were positively related (adjusted *R*^2^ = 0.688, *p* < .001). For protein-coding genes, observed rates of substitutions/site (Table 1) were not strongly correlated with the rates calculated from other songbird (Aves: Passeriformes) mitochondrial genomes (*R*^2^=0.10, *p*=.34; Nguyen and Ho 2016). For rRNAs (12S and 16S), we recovered greater than 90% identical nucleotides for each respective gene region. Among commonly used genes (CytB, COI, ND2), we recover much lower proportions of identical nucleotides across taxa (75.3, 77.1, and 63.6%, respectively), suggesting high mutation rates.

**Table 1.** Genetic elements used for gene tree analyses, and associated statistics.

Gene region	Align. length	% Identical nucleotide	% Pairwise identity	ΔML score	Nucl. Subs./Site	^a^Variable residues	^b^Relative rate	^b^Adjusted *R*^2^
12s	978	90.4	97.6	1.673	0.109	–	0.323	0.614
16s	1604	90.0	96.9	1.014	0.116	–	0.398	0.699
ATP6	684	73.5	92.1	17.147	0.307	16/227	1.014	0.780
ATP8	168	73.2	91.9	9.905	0.298	7/53	0.914	0.609
CR	1243	65.5	88.8	1.808	0.422	–	1.344	0.698
CO1	1551	77.1	92.8	2.576	0.262	9/516	0.888	0.843
CO2	684	73.7	91.4	0.732	0.303	14/227	1.111	0.833
CO3	784	75.6	92.3	3.241	0.281	11/261	0.869	0.729
CytB	1143	75.3	92.6	4.985	0.287	33/380	–	–
ND1	978	68.8	89.4	9.005	0.355	26/325	1.389	0.874
ND2	1038	63.6	88.3	4.216	0.398	70/346	1.376	0.833
ND3	351	68.1	90.0	3.261	0.368	16/116	1.495	0.828
ND4	1378	70.4	91.2	11.035	0.331	45/459	1.124	0.908
ND4L	297	73.1	92.6	3.525	0.296	8/99	0.883	0.622
ND5	1818	70.6	91.3	2.229	0.332	84/605	0.974	0.885
ND6	519	68.0	90.8	9.045	0.372	33/172	0.988	0.764

^a^ Number of variable and total amino acid residues in the protein open reading frame.

^b^ Relative rates of sequence evolution for each gene relative to CytB, based on regression analyses of pairwise divergence between all samples and associated *R*^2^ value.

**Figure 1.**
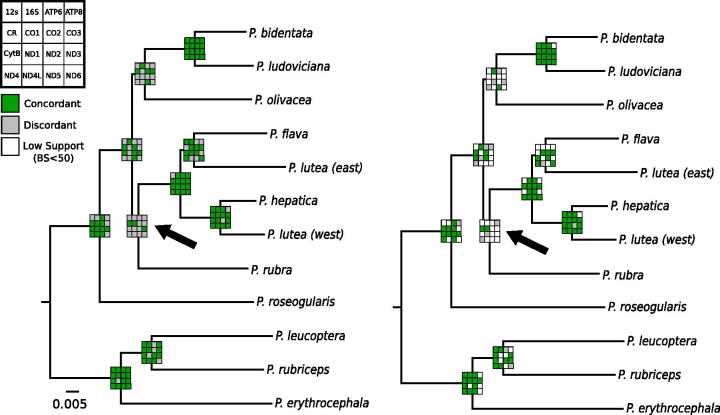
Maximum likelihood mitogenome tree, which is topologically identical to the nuclear DNA species tree from Manthey et al. (2016), showing concordance of individual gene trees at each node. Concordance between the mitogenome tree and each gene tree is shown (A) without any limit on bipartition support (BS ≥ 0%) and B) with a lower limit on support necessary to be included in the comparison (BS ≥ 50%). The black arrow shows the node that had low bootstrap support in the concatenated mitogenome tree (BS = 57%).

When comparing the mitogenome tree to the nuclear species tree, we found the RAxML mitogenome tree (Figure 1) was topologically identical to the nuclear species tree, with all but one node having high (>96%) bootstrap support (*P. rubra* sister to *P. lutea* + *P. hepatica* + *P. flava* clade had 57% BS support). When comparing each estimated gene tree from the 16 non-tRNA elements to the concatenated mitogenome tree, we found that no single gene was topologically identical to the mitogenome tree. The gene tree most concordant with the nuclear species tree and mitogenome tree topology was the Control Region (concordant at 9 of 10 nodes; Figure 1(A)), with a handful of other genes concordant at eight nodes (CO1, CO2, 16S, ND5, and CytB). We found the difference in likelihood scores between the constrained and unconstrained gene trees to be most similar for CO2 (ΔML = 0.732; Table 1), and greatest for ATP6 (ΔML = 17.147).

## Discussion

We investigated patterns of topological concordance with the nuclear species tree identified using thousands of loci (Manthey et al. 2016) but failed to recover a single mitochondrial element that was completely concordant with the species tree (which was topologically identical to the concatenated mitogenome tree). Support varied across the dataset, with as few as three gene trees showing the same branching pattern at a given node, while all genes showed the same bipartition at a single node (Figure 1(A)). The number of parsimony informative sites (i.e. phylogenetic informativeness) may cause unresolved nodes in mtDNA gene trees. However, low bootstrap support at particular nodes on individual gene trees may give a false impression of topological concordance when in fact the relationship is relatively unsupported. In fact, many of the individual gene trees had low bootstrap support at internal nodes, and therefore, it is likely they did not greatly contribute to the overall mitogenome tree (Figure 1(B)).

Our estimate of the relative intergenic divergence rate of ND2 to CytB (1.37; Table 1) for *Piranga* is qualitatively similar to the relative intrageneric rate of divergence for a subset of New World birds (1.27) by Smith and Klicka (2010) for the same genetic markers. Nucleotide conservation across the mitogenome varied by gene. For rRNAs, we recovered greater than 90% identical nucleotides, which was not surprising, given the necessary role rRNA plays in protein synthesis. We also found that differences in the likelihood scores between constrained and unconstrained gene trees for the two rRNAs were the highest observed. Among genes commonly used in avian systematics (CytB, COI, ND2), we recover lower proportions of identical nucleotides across taxa (75.3, 77.1, and 63.6%, respectively). Sequence variation is necessary for a gene region to be phylogenetically informative, without variability reconstruction of evolutionary history would fail. Since CytB, COI, and ND2 all have increased rates of substitution, they should have improved utility for phylogenetic reconstruction.

Within the mitogenome, we find evidence for variable relationships depending on which region of the mitogenome is considered. However, regardless of the underlying cause of gene tree discordance within the mitogenome, when considering the entire mitogenome, as one linkage group, we recovered an identical topology to the nuclear species tree topology. Altogether, these findings point to a broader issue in phylogenetics, in which researchers erroneously assume different regions of the mitogenome represent separate, independent ‘genes.’ This study, along with published examples of mito-nuclear discordance and discordance between individual mtDNA regions, reiterates the fact that not recognizing the mitogenome as a single linkage group could lead to a multitude of phylogenetic and systematic errors.
